# Novel indicator for erectile dysfunction: the CALLY index, evidence from data of NHANES 2001-2004

**DOI:** 10.3389/fendo.2025.1527506

**Published:** 2025-03-03

**Authors:** Dongli Huang, Hang Wu, Yanhua Huang

**Affiliations:** ^1^ Bishan Hospital of Chongqing Medical University (Bishan Hospital of Chongqing), Chongqing, China; ^2^ Department of Infectious Diseases, Chongqing University Three Gorges Hospital, Chongqing, China

**Keywords:** the C-reactive protein-albumin-lymphocyte, erectile dysfunction, national health and nutrition examination survey, cross-sectional study, inflammation marker

## Abstract

**Purpose:**

This study explored the association between the C-reactive protein-albumin-lymphocyte (CALLY) index and erectile dysfunction (ED).

**Patients and methods:**

Data from 2,128 participants in the 2001–2004 National Health and Nutrition Examination Survey (NHANES) were analyzed and classified into ED and non-ED groups.Additionally, a separate analysis of complete erectile dysfunction was conducted.A weighted multiple logistic regression model was used to assess the association between CALLY and ED, while smooth curve fitting was applied to explore their linear relationship.ROC analysis was conducted to compare the predictive accuracy (AUC) of CALLY, Systemic Inflammation Response Index (SIRI), Systemic Immune-Inflammation Index (SII), Aggregate Index of Systemic Inflammation (AISI), Neutrophil-to-Lymphocyte Ratio (NLR), Platelet-to-Lymphocyte Ratio (PLR), and the product of platelet count and neutrophil count (PPN) for ED.

**Results:**

After adjustment, Ln-CALLY was negatively associated with ED (OR = 0.77, 95% CI: 0.69–0.85, p < 0.0001) and complete ED (OR = 0.88, 95% CI: 0.78–1.00, p = 0.0450).The highest Ln-CALLY tertile (Q3) was associated with a significantly lower risk of ED compared to Q1 (OR = 0.40, 95% CI: 0.30–0.55, p < 0.0001).A similar trend was observed for complete ED (OR = 0.57, 95% CI: 0.38–0.85, p = 0.006).Curve fitting revealed a negative correlation between CALLY and both types of ED.Subgroup analysis confirmed the consistent and independent association.CALLY exhibited superior predictive performance for ED (AUC = 0.6512) and complete ED (AUC = 0.6237) compared to other markers.

**Conclusion:**

Higher CALLY levels were linked to a reduced ED risk and proved a superior predictor compared to other inflammatory markers.

## Introduction

Erectile dysfunction is a common disease of the male reproductive system that seriously affects the quality of life of both the patient and their partner, which has a prevalence of 19-52% in the world and increases with age (1). ED is a multifactorial disease associated with various factors, including injury, endothelial dysfunction, smooth muscle issues, infection, and oxidative stress (2). Evidence indicates systemic inflammation contributes to endothelial dysfunction, which is linked to various vascular diseases (3, 4). Penile arteries are small and sensitive; therefore, ED may significantly indicate systemic endothelial dysfunction. Consequently, considerable research has been directed toward exploring the relationship between inflammation and ED.

In clinical and previous studies, hematological indicators are frequently used to reflect patients’ inflammatory levels, nutritional status, and immune function. C-reactive protein (CRP) is a common clinical marker used to reflect the inflammatory status of patients with ED (5). Serum albumin has long been used as a marker of clinical nutritional status, with low albumin serving as an independent risk factor for ED. Lymphocyte count is a conventional biomarker that reflects immune function (6, 7). The C-reactive protein-albumin-lymphocyte (CALLY) index, developed by Hiroya Iida et al., is a novel immunity and nutrition scoring system calculated by multiplying the albumin level by the lymphocyte count and dividing by the C-reactive protein level (8). Previous studies have investigated the relationship between CALLY and the prognosis of patients with colorectal cancer, hepatocellular carcinoma, and myocardial infarction (8–11). Similarly, a cross-sectional study showed that the CALLY index was negatively correlated with cardiocirculatory syndrome (12). These studies show that, compared with traditional immune indicators, the CALLY index has excellent predictive power for various diseases. However, studies on the correlation between the CALLY index and ED are still limited. Using data from the National Health and Nutrition Examination Survey (NHANES), we investigated the potential independent association between the CALLY index and ED to address gaps in understanding ED risk factors and predictors and to guide the development of effective preventive measures.

## Materials and methods

We performed a cross-sectional analysis using NHANES data from 2001 to 2004. This comprehensive program, administered by the National Center for Health Statistics, is a vital resource for assessing the health and nutritional status of the US population.NHANES includes detailed interviews covering demographic characteristics, socioeconomic status, dietary practices, and various health aspects, supplemented by thorough examinations conducted by qualified healthcare professionals, including medical tests and blood marker assessments. All participants in the NHANES study program provided informed consent, and the NCHS Research Ethics Review Board approved the program. Detailed information about the NHANES survey is publicly available.

### Data and study participants

The National Health and Nutrition Examination Survey (NHANES) is a major initiative of the Centers for Disease Control and Prevention (CDC) designed to assess the health and nutritional status of the U.S. population. NHANES uses a multistage probability sampling design to collect a representative sample of the noninstitutionalized civilian population in the United States. All participants in the NHANES study protocol provided informed consent, and the protocol was approved by the NCHS Research Ethics Review Board. Further details about the NHANES survey can be found on the official website: https://www.cdc.gov/nchs/nhanes/index.htm.

**Figure 1:**
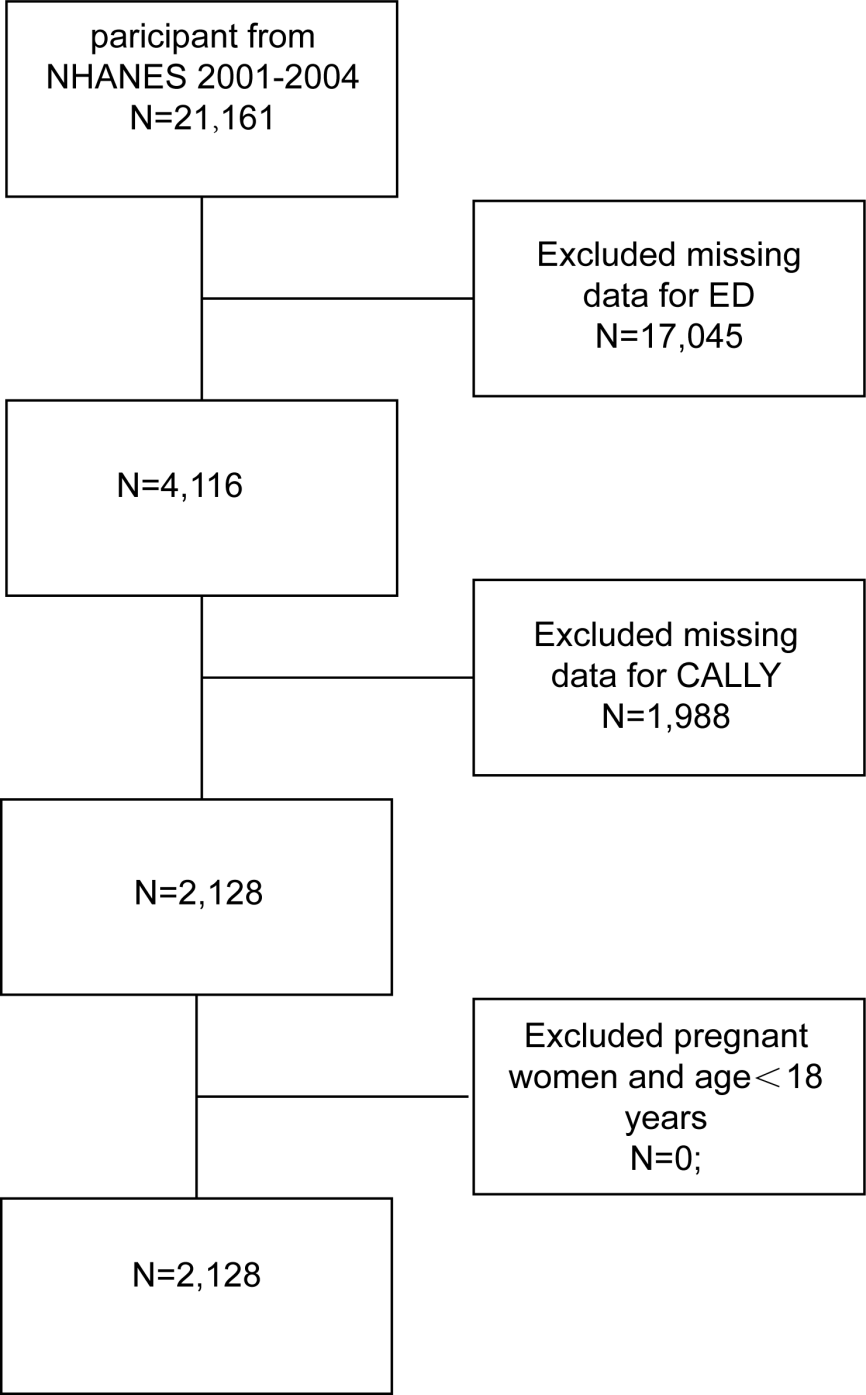
Flowchart of participant selection. NHANES, National Health and Nutrition Examination Survey; CALLY, C-reactive protein-albumin-lymphocyte; ED, erectile dysfunctioncholesterol ratio.

For our study, we utilised data from two NHANES cycles (2001-2002 and 2003-2004) as ED information was only available for these years. The initial sample consisted of 21,161 participants. We then applied the following inclusion and exclusion criteria:(1) Participants with incomplete ED assessments, as assessed through the “Body Measures” and “Prostate Conditions” questionnaires, were excluded (n=17,045). (2) Participants lacking the necessary data for calculating the C-reactive protein-albumin-lymphocyte (CALLY) index, specifically CRP, albumin, and lymphocyte data, were excluded (n=1,988). (3) Participants under the age of 18 years were excluded (n=0). As a result, the final study population consisted of 2,128 participants. The detailed selection process, including all exclusions, is shown in **Figure 1**.

### Assessment of C-reactive protein-albumin-lymphocyte index

The blood cell count analysis results, including neutrophils, platelets, monocytes, and lymphocytes, obtained using the Kurt HMX blood analyzer, are reported in standardized units per microliter × 10³ cells. Our primary focus is the CALLY index, a novel indicator derived from albumin levels, lymphocyte counts, and CRP values. The CALLY index is calculated explicitly as [(albumin in g/L) × (lymphocyte count in 1000 cells/μL)]/(CRP in mg/dL), offering a comprehensive assessment of inflammation and nutritional status.

### Assessment of ED

Interviews were conducted in private rooms at the MEC using the Audio Computer-Assisted Self-Interview (ACASI) method.ED self-assessment, used as the dependent variable, was measured with a question from the Massachusetts Male Aging Study (13): ‘How would you describe your ability to get and maintain an erection sufficient for satisfactory sexual intercourse?’Response options included ‘always or almost always can,’ ‘usually can,’ ‘sometimes can,’ and ‘never can.’For this analysis, ED was defined as participants who answered ‘sometimes able’ or ‘never able’ to maintain an erection. Respondents who answered ‘always or almost always able’ or ‘usually able’ to maintain an erection were classified as not having ED. In this study, complete erectile dysfunction was defined as participants who answered ‘never able’ to maintain an erection.

### Covariates of interest

In this study, the covariates of interest included age, race, education level, marital status, family poverty income ratio (PIR), BMI, physical activity (vigorous/moderate), smoking status, alcohol consumption, diabetes, hypertension, hypercholesterolemia, and cardiovascular disease(CVD). Physical activity was categorized as vigorous (yes/no) or moderate (yes/no). Participants who reported smoking at least 100 cigarettes in their lifetime and were smoking at the time of the survey were classified as current smokers. Former smokers were defined as those who had smoked at least 100 cigarettes in their lifetime but were not smoking at the time of the survey. Additionally, men who had smoked fewer than 100 cigarettes in their lifetime were classified as non-smokers. A non-drinker was a participant who answered ‘no’ to having consumed at least 12 alcoholic beverages in their lifetime or any single year. In this study, men who answered ‘yes’ to having consumed 12 alcoholic drinks in their lifetime or any single year but had not consumed any in the past 12 months were classified as former drinkers. Participants who answered ‘yes’ to consuming 12 alcoholic beverages in their lifetime or any single year and had consumed at least one in the past 12 months were classified as current drinkers. Participants were considered diabetic if they had a physician’s diagnosis of diabetes, a fasting blood glucose level ≥ 7.0 mmol/L, glycated hemoglobin (HbA1c) ≥ 6.5%, or were using glucose-lowering medication or insulin, according to the American Diabetes Association (ADA) criteria. Participants were classified as hypertensive if they had a history of hypertension, were taking antihypertensive medications, or had a systolic blood pressure ≥ 130 mmHg or diastolic blood pressure ≥ 80 mmHg, according to the 2017 American College of Cardiology (ACC) and American Heart Association (AHA) guidelines. Participants were considered hypercholesterolemic if their total cholesterol level was > 5.2 mmol/L or their low-density lipoprotein cholesterol (LDL-C) level was > 3.4 mmol/L, according to the National Cholesterol Education Program (NCEP) Adult Treatment Panel III (ATP III) guidelines.CVD was defined as participants with a prior diagnosis of congestive heart failure, coronary heart disease, angina pectoris, stroke, or heart attack.

### Statistical analysis

In this study, the multistage design of NHANES was accounted for in all statistical analyses by selecting appropriate sampling weights, strata, and primary sampling units.Continuous variables were expressed as weighted means and standard errors (SE), and categorical variables were expressed as weighted proportions in the baseline characteristics table. We observed that the CALLY data exhibited skewness, necessitating the application of the natural logarithm (Ln) to the values prior to statistical analysis (**Supplementary Figures 1**, **2**). The Kruskal Wallis rank sum test was used for continuous variables and the chi-square test for categorical variables to assess differences between participants with different CALLY tertiles. The relationship between CALLY and ED prevalence was analyzed using multivariate logistic regression. Three models were used: Model 1 was unadjusted; Model 2 was adjusted for age and race; and Model 3 was adjusted for age, race, BMI, education level, marital status, family PIR, smoking status, alcohol consumption, vigorous and moderate activity, diabetes, hypertension, high cholesterol, and CVD. Subgroup analyses explored the association between CALLY and ED across various subgroups. Smooth curve fitting was applied to examine the nonlinear relationship between CALLY and ED. The Receiver Operating Characteristic (ROC) curve and the area under the curve (AUC) were used to evaluate the predictive ability of CALLY and other inflammatory markers (SIRI, SII, AISI, NLR, PLR, and PPN) for ED. A p-value < 0.05 was considered statistically significant. All statistical analyses were conducted using EmpowerStats (http://www.empowerstats.com, X&Y Solutions, Inc.) and the R statistical software package (http://www.R-project.org; The R Foundation).

## Results

### Characteristics of study participants

A total of 2,128 participants were included in our study, with 709 in T1, 709 in T2, and 710 in T3 of the three CALLY groups **Table 1** shows the baseline demographic and clinical characteristics of the participants. Of the participants, 648 (30.45%) were diagnosed with ED, and 268 (12.59%) had complete ED. No statistically significant differences in education level were observed between groups (P > 0.05). At the same time, all other baseline covariates showed statistically significant differences between CALLY groups (P < 0.05).

**Table 1:** Baseline characteristics of NHANES participants, 2001–2004.

Variables	Tertiles of Ln-CALLY			*P value*
	T1	T2	T3	
N	709	709	710	
CALLY	152.63 ± 79.49	529.57 ± 156.65	2444.46 ± 4079.47	<0.001
TG, (mg/dL)	143.07 ± 84.27	142.82 ± 81.06	130.68 ± 89.79	<0.001
LDL, (mmol/L)	3.08 ± 0.65	3.02 ± 0.60	2.96 ± 0.57	0.008
HDL, (mmol/L)	1.20 ± 0.33	1.24 ± 0.32	1.32 ± 0.36	<0.001
TC, (mmol/L)	5.18 ± 1.11	5.16 ± 1.04	5.00 ± 1.03	0.003
CRP, (mg/dL)	0.88 ± 1.50	0.19 ± 0.08	0.06 ± 0.05	<0.001
ALB, (g/L)	41.83 ± 3.11	43.50 ± 2.76	44.80 ± 3.04	<0.001
LYM, (1000 cells/dL)	1.84 ± 0.64	2.10 ± 0.74	2.45 ± 4.05	<0.001
NLR	2.74 ± 1.40	2.17 ± 0.9	1.92 ± 0.83	<0.001
PLR	148.29 ± 58.46	127.67 ± 49.62	121.71 ± 45.07	<0.001
SII	680.02 ± 402.74	540.46 ± 488.42	473.97 ± 248.44	<0.001
AISI	419.83 ± 347.96	310.08 ± 237.29	266.70 ± 180.26	<0.001
SIRI	1.66 ± 1.20	1.24 ± 0.72	1.07 ± 0.62	<0.001
BMI, n (%)				<0.001
<25	133 (18.76%)	172 (24.26%)	311 (43.80%)	
>=25, <30	278 (39.21%)	302 (42.60%)	283 (39.86%)	
>=30	298 (42.03%)	235 (33.15%)	116 (16.34%)	
Age, n (%)				<0.001
<50	293 (41.33%)	336 (47.39%)	476 (67.04%)	
>=50	416 (58.67%)	373 (52.61%)	234 (32.96%)	
Race, n (%)				<0.001
Mexican American	119 (16.78%)	157 (22.14%)	136 (19.15%)	
Other Hispanic	18 (2.54%)	22 (3.10%)	19 (2.68%)	
Non-Hispanic White	398 (56.14%)	386 (54.44%)	389 (54.79%)	
Non-Hispanic Black	156 (22.00%)	116 (16.36%)	132 (18.59%)	
Other Race	18 (2.54%)	28 (3.95%)	34 (4.79%)	
Education, n (%)				0.068
Under high school	199 (28.07%)	191 (26.94%)	177 (24.93%)	
Hight school or equivalent	188 (26.52%)	191 (26.94%)	159 (22.39%)	
College graduate or above	322 (45.41%)	327 (46.12%)	374 (52.68%)	
Marital Status, n (%)				0.002
Married or living with partner	475 (67.00%)	514 (72.50%)	454 (63.94%)	
Living alone	234 (33.00%)	195 (27.50%)	256 (36.06%)	
PIR, n (%)				0.021
<1.3	178 (25.11%)	136 (19.18%)	180 (25.35%)	
>=1.3, <3.5	303 (42.74%)	321 (45.28%)	281 (39.58%)	
>=3.5	228 (32.16%)	252 (35.54%)	249 (35.07%)	
CVD				<0.001
Yes	126 (17.77%)	102 (14.39%)	65 (9.15%)	
No	583 (82.23%)	607 (85.61%)	645 (90.85%)	
Diabetes, n (%)				<0.001
Yes	148 (20.87%)	99 (13.96%)	60 (8.45%)	
No	561 (79.13%)	610 (86.04%)	650 (91.55%)	
Drink, n (%)				<0.001
Current drinkers	472 (66.57%)	510 (71.93%)	545 (76.76%)	
Nondrinkers	57 (8.04%)	35 (4.94%)	51 (7.18%)	
Former drinkers	180 (25.39%)	164 (23.13%)	114 (16.06%)	
Hypertension, n (%)				<0.001
Yes	468 (66.01%)	440 (62.06%)	300 (42.25%)	
No	241 (33.99%)	269 (37.94%)	410 (57.75%)	
Vigorous activity, n (%)				<0.001
Yes	154 (21.72%)	221 (31.17%)	290 (40.85%)	
No	555 (78.28%)	488 (68.83%)	420 (59.15%)	
Moderate activity, n (%)				0.003
Yes	360 (50.78%)	394 (55.57%)	409 (57.61%)	
No	349 (49.22%)	315 (44.43%)	301 (42.39%)	
Smoke, n (%)				<0.001
Current smokers	199 (28.07%)	190 (26.80%)	188 (26.48%)	
Nonsmokers	242 (34.13%)	291 (41.04%)	330 (46.48%)	
Former smokers	268 (37.80%)	228 (32.16%)	192 (27.04%)	
Hyperlipidemia, n (%)				<0.001
Yes	512 (72.21%)	481 (67.84%)	395 (55.63%)	
No	197 (27.79%)	228 (32.16%)	315 (44.37%)	
Erectile dysfunction, n (%)				<0.001
Yes	305 (43.02%)	221 (31.17%)	122 (17.18%)	
No	404 (56.98%)	488 (68.83%)	588 (82.82%)	
Complete erectile dysfunction, n (%)				<0.001
Yes	124 (17.49%)	97 (13.68%)	47 (6.62%)	
No	585 (82.51%)	612 (86.32%)	663 (93.38%)	

Categorized according to CALLY tertiles. CALLY, C-reactive protein-albumin-lymphocyte; BMI, body mass index; SII, systemic Immune-Inflammation Index.

### Association between CALLY and ED

**Figure 2:**
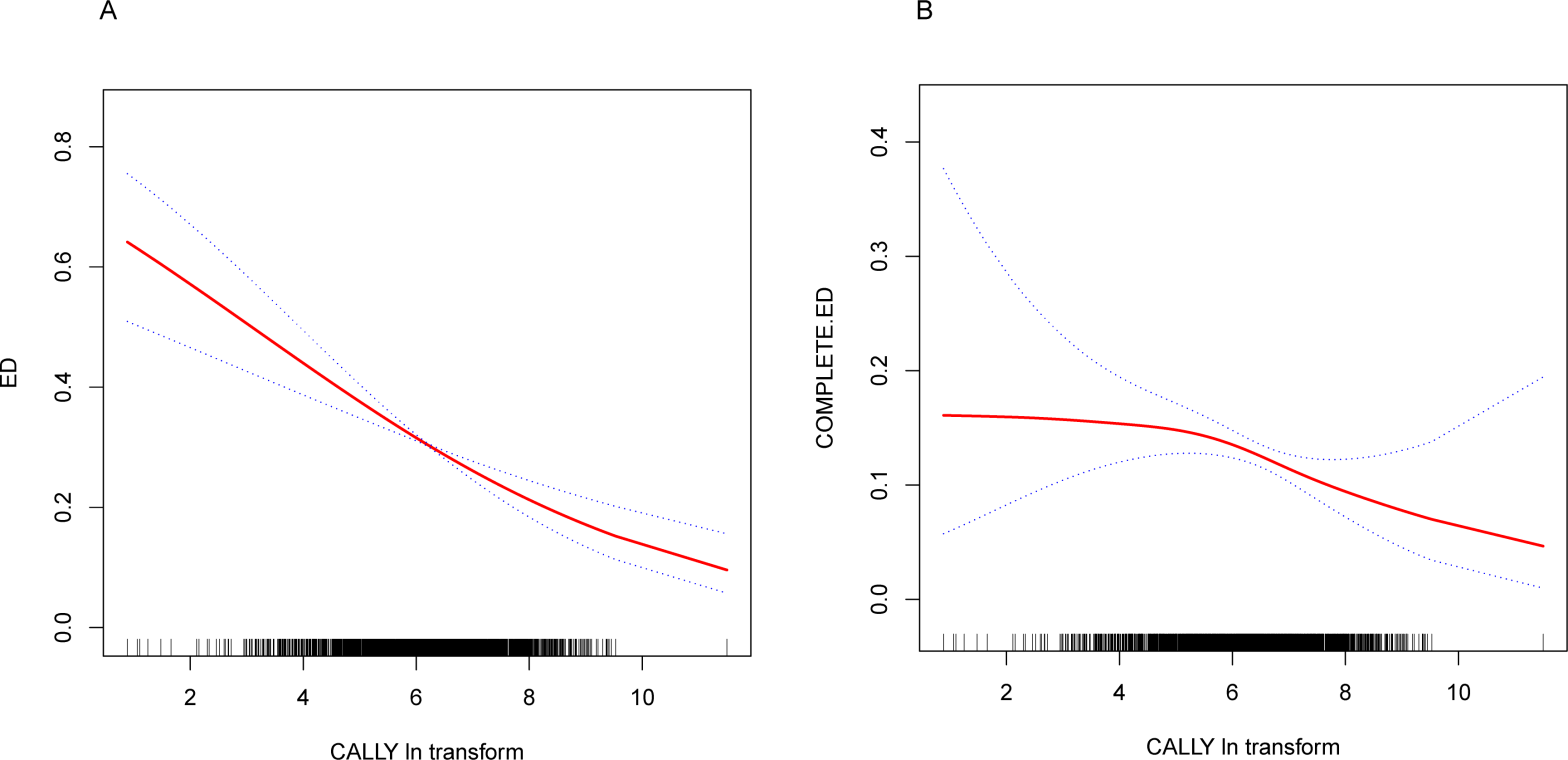
**(A)** Smooth curve fitting diagram of Ln-AISI and erectile dysfunction in fully adjusted models; **(B)** Smooth curve fitting diagram of Ln-AISI and complete erectile dysfunction in fully adjusted models.

Weighted multivariate logistic regression was used to examine the relationship between CALLY and ED in the crude model, Model 1, and the fully adjusted model. Details are presented in **Table 2**, which showed a significant negative correlation between Ln-CALLY and ED and complete ED across all models. In the fully adjusted model (Model 3), each one-unit increase in Ln-CALLY was associated with a 23% reduction in the risk of ED (OR = 0.77, 95% CI: 0.69–0.85, p < 0.0001). The risk of complete ED decreased by 12% (OR = 0.88, 95% CI: 0.78–1.00, p = 0.0450). The continuous Ln-CALLY variable was converted into a categorical variable by dividing it into three tertiles. In Model 3, individuals in the highest tertile (T3) had a 60% lower risk of ED compared to those in the lowest tertile (T1) (OR = 0.40, 95% CI: 0.30–0.55, p < 0.0001). This trend was consistent for complete ED, with an OR of 0.57 (95% CI: 0.38–0.85, p = 0.0066). Smooth curve fitting analysis revealed a negative linear correlation between CALLY and both ED and complete ED (**Figure 2**).

**Table 2:** Multiple logistic regression analysis Ln-CALLY vs erectile dysfunction.

	Crude Model OR 95% CI	*P-*value	Model 1 OR 95% CI	*P*-value	Model 2 OR 95% CI	*P*-value
Ln-CALLY VS ED	0.66 (0.61, 0.71)	<0.0001	0.74 (0.68, 0.81)	<0.0001	0.77 (0.69, 0.85)	<0.0001
Stratified by Ln-CALLY tertiles						
T1	ref		ref		ref	
T2	0.60 (0.48, 0.75)	<0.0001	0.58 (0.45, 0.75)	<0.0001	0.64 (0.49, 0.84)	0.0012
T3	0.27 (0.22, 0.35)	<0.0001	0.38 (0.29, 0.51)	<0.0001	0.40 (0.30, 0.55)	<0.0001
*P* for trend	10.59 (0.53, 0.65)	<0.0001	0.67 (0.59, 0.75)	<0.0001	0.68 (0.60, 0.78)	<0.0001
Ln-CALLY VS CED	0.72 (0.66, 0.80)	<0.0001	0.84 (0.75, 0.94)	0.0027	0.88 (0.78, 1.00)	0.0450
Stratified by Ln-CALLY tertiles						
T1	Ref		Ref		Ref	
T2	0.75 (0.56, 1.00)	0.0485	0.80 (0.59, 1.09)	0.1585	0.93 (0.67, 1.30)	0.6695
T3	0.33 (0.23, 0.48)	<0.0001	0.54 (0.37, 0.79)	0.0013	0.57 (0.38, 0.85)	0.0066
*P* for trend	0.65 (0.57, 0.75)	<0.0001	0.78 (0.67, 0.91)	0.0014	0.81 (0.69, 0.95)	0.0116

ED, erectile dysfunction; CED, complete erectile dysfunction; CVD, cardiovascular disease.

OR, odds ratio.

95% CI, 95% confidence interval.

Model 1, no covariates were adjusted

Model 2, adjusted for age, and race.

Model 3: age, race, BMI, education, marital status, PIR, hyperlipidemia, diabetes, drink, hypertension, Vigorous activity, Moderate activity, CVD, smoke.

### Subgroup analysis

Subgroup analyses were conducted to determine whether the association between Ln-CALLY and ED and complete ED was consistent across different strata. As shown in **Table 3**, all subgroups, including age, BMI, hypertension, diabetes mellitus, high cholesterol, vigorous activity, moderate activity, and CVD, did not affect the negative correlation between CALLY and ED or complete ED (p > 0.05 for all interactions).In certain strata, the negative correlations were statistically significant and stable. For instance, among participants over 50 years of age, each unit increase in Ln-CALLY was associated with a 27% reduction in the likelihood of developing ED (OR = 0.73, 95% CI: 0.65–0.83, p = 0.029) and a 12% reduction in the possibility of creating complete ED (OR = 0.88, 95% CI: 0.77–1.00, p = 0.0431). Detailed results of this analysis are presented in **Table 3**.

**Table 3.1:** Subgroup analysis for the association between CALLY and ED.

Character	OR 95% CI	*P*-value	P for interaction
BMI			0.6375
Blow 25	0.82 (0.68, 1.00)	0.0514	
25-29.9	0.73 (0.63, 0.85)	<0.001	
≥30	0.78 (0.64, 0.95)	0.0141	
PIR			0.1639
Blow 1.3	0.79 (0.66, 0.95)	0.0142	
1.3-3.5	0.83 (0.71, 0.96)	0.0143	
Over 3.5	0.66 (0.55, 0.80)	<0.0001	
Drink			0.6755
Current drinkers	0.78 (0.69, 0.88)	<0.0001	
Nondrinkers	0.79 (0.53, 1.18)	0.2493	
Former drinkers	0.70 (0.57, 0.86)	<0.0001	
Hypertension			0.4893
Yes	0.74 (0.66, 0.84)	<0.0001	
No	0.80 (0.68, 0.94)	0.0054	
Diabetes			0.3511
Yes	0.84 (0.66, 1.07)	0.1647	
No	0.74 (0.66, 0.83)	<0.0001	
Vigorous activity			0.2452
Yes	0.88 (0.69, 1.12)	0.2869	
No	0.75 (0.67, 0.84)	<0.0001	
Moderate activity			0.5522
Yes	0.79 (0.68, 0.91)	0.0016	
No	0.74 (0.65, 0.85)	<0.0001	
Marital Status			0.4122
Married or living with partner	0.74 (0.66, 0.84)	<0.0001	
Living alone	0.81 (0.68, 0.96)	0.0173	
Smoke			0.8461
Current smokers	0.76 (0.63, 0.93)	0.0064	
Nonsmokers	0.77 (0.65, 0.92)	0.0042	
Former smokers	0.72 (0.61, 0.86)	0.0002	
Age			0.2036
<50	0.85 (0.70, 1.04)	0.1137	
>=50	0.73 (0.65, 0.83)	<0.0001	
CVD			0.9589
Yes	0.76 (0.59, 0.98)	0.0324	
No	0.76 (0.69, 0.85)	<0.0001	
Hyperlipidemia			0.1058
Yes	0.81 (0.72, 0.92)	0.0009	
No	0.69 (0.58, 0.81)	<0.0001	

**Table 3.2:** Subgroup analysis for the association between CALLY and complete ED.

Character	OR 95% CI	*P*-value	P for interaction
BMI			0.5925
Blow 25	0.98 (0.77, 1.23)	0.8450	
25-29.9	0.84 (0.70, 1.01)	0.0612	
≥30	0.88 (0.67, 1.14)	0.3300	
PIR			0.3474
Blow 1.3	0.87 (0.70, 1.09)	0.2251	
1.3-3.5	0.97 (0.81, 1.16)	0.7236	
Over 3.5	0.77 (0.59, 0.99)	0.0457	
Drink			0.1097
Current drinkers	0.93 (0.79, 1.10)	0.4077	
Nondrinkers	0.48 (0.26, 0.89)	0.0205	
Former drinkers	0.90 (0.74, 1.09)	0.2843	
Hypertension			0.7068
Yes	0.89 (0.77, 1.03)	0.1247	
No	0.85 (0.69, 1.04)	0.1208	
Diabetes			0.8287
Yes	0.90 (0.72, 1.14)	0.4028	
No	0.88 (0.76, 1.01)	0.0720	
Vigorous activity			0.6934
Yes	0.96 (0.63, 1.46)	0.8548	
No	0.88 (0.78, 1.00)	0.0526	
Moderate activity			0.0518
Yes	1.00 (0.83, 1.20)	0.9853	
No	0.78 (0.66, 0.93)	0.0042	
Marital Status			0.7717
Married or living with partner	0.89 (0.77, 1.02)	0.1000	
Living alone	0.85 (0.69, 1.06)	0.1455	
Smoke			0.9456
Current smokers	0.92 (0.67, 1.28)	0.6234	
Nonsmokers	0.88 (0.70, 1.09)	0.2369	
Former smokers	0.87 (0.72, 1.03)	0.1126	
Age			0.8182
<50	0.92 (0.62, 1.37)	0.6828	
>=50	0.88 (0.77, 1.00)	0.0431	
CVD			0.7135
Yes	0.91 (0.73, 1.13)	0.4060	
No	0.87 (0.75, 1.00)	0.0579	
Hyperlipidemia			0.1279
Yes	0.94 (0.81, 1.10)	0.4275	
No	0.78 (0.64, 0.94)	0.0112	

### CALLY had a more vital predictive ability for ED and complete ED than other Inflammation marks

**Figure 3:**
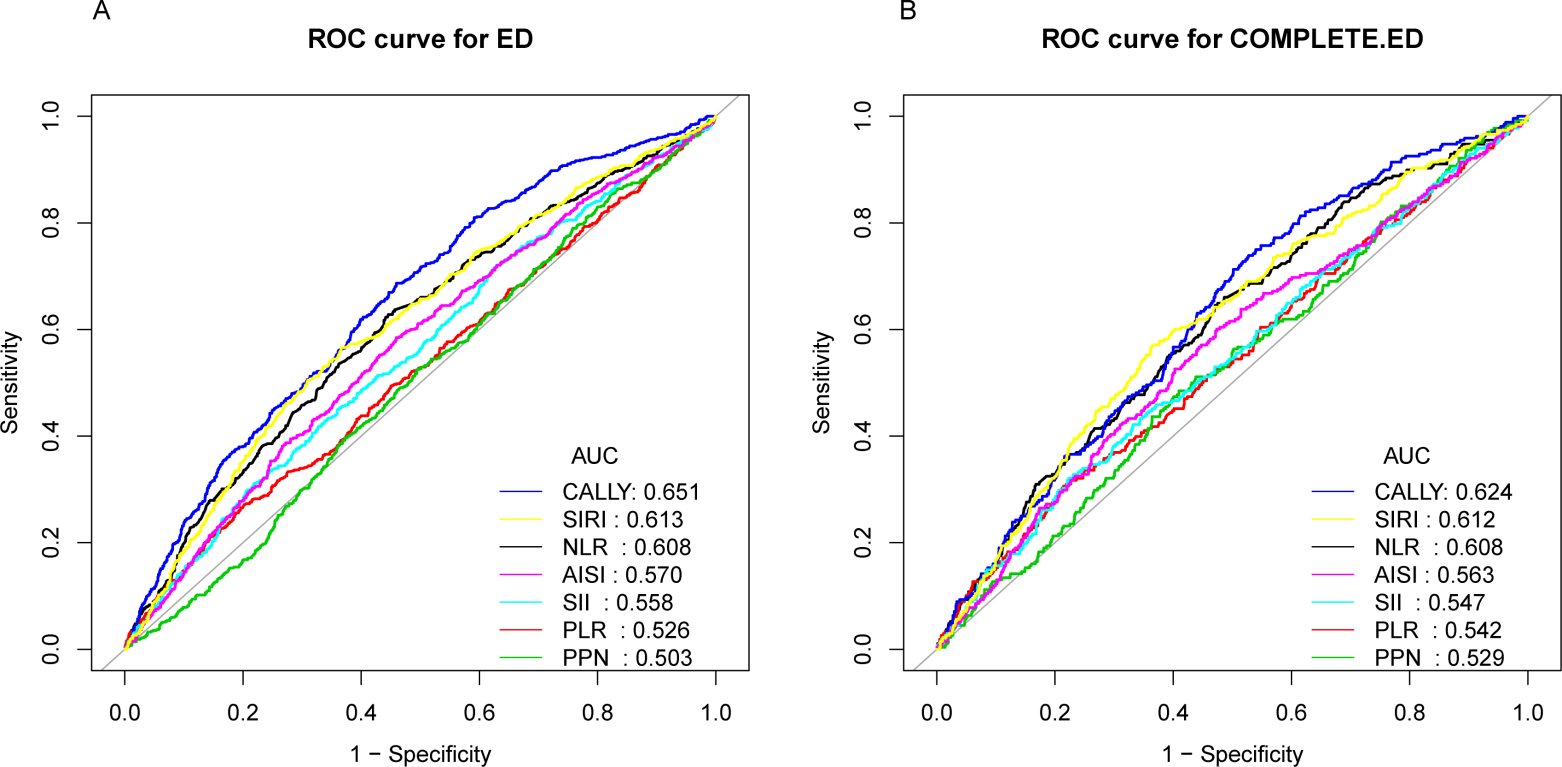
ROC curve and AUC value of seven inflammatory indicators(CALLY, SIRI, NLR, AISI, SII, PLR, PPN) for dignosing erectile dysfunction and complete erectile dysfunction. **(A)** seven inflammatory indicators are assessed to identify erectile dysfunction. **(B)** seven inflammatory indicators are assessed to identify complete erectile dysfunction.

The area under the curve (AUC) was calculated to compare the predictive accuracy of CALLY with other inflammatory indicators (NLR, PLR, PPN, SII, AISI, SIRI) for ED and complete ED. **Figure 3** shows the ROC curve used to evaluate the predictive performance of CALLY and other inflammatory markers for ED and complete ED. **Table 4** presents the AUC value (95% CI) for diagnosing ED: CALLY: 0.6512 (0.6263–0.6762).CALLY demonstrated the highest AUC compared to other inflammatory markers, with a statistically significant difference (p < 0.05). The AUC of CALLY was also the highest for diagnosing complete ED. **Table 4** includes additional details, such as cut-off values, sensitivity, and specificity. For instance, the optimal cut-off value for CALLY in predicting ED was 556.79 (specificity: 0.5405, sensitivity: 0.6867). These results suggest that CALLY may be a superior predictor of ED and complete ED compared to other inflammatory markers.

**Table 4:** Comparison of AUC values between CALLY and other Inflammation marks.

Test	AUC	95%CI low	95%CI upp	Best threshold	Specificity	Sensitivity	*P* for different in AUC
ED							
CALLY	0.6512	0.6263	0.6762	556.7857	0.5405	0.6867	reference
NLR	0.6076	0.5813	0.6338	2.0607	0.5541	0.6281	0.0037
PLR	0.5259	0.4987	0.5532	162.4265	0.7980	0.2716	<0.0001
PPN	0.5029	0.4766	0.5291	664.5500	0.2419	0.7901	<0.0001
SII	0.5576	0.5309	0.5843	687.0000	0.7804	0.3117	<0.0001
AISI	0.5698	0.5432	0.5963	263.7420	0.5405	0.5849	<0.0001
SIRI	0.6126	0.5864	0.6387	1.2372	0.6338	0.5664	0.0124
CED							
CALLY	0.6237	0.5897	0.6578	597.1825	0.4726	0.7388	reference
NLR	0.6081	0.5723	0.6439	2.0755	0.5290	0.6455	0.4449
PLR	0.5422	0.5040	0.5803	162.0820	0.7855	0.3060	0.0005
PPN	0.5288	0.4923	0.5653	858.6000	0.5903	0.4851	0.0005
SII	0.5469	0.5090	0.5848	701.7647	0.7769	0.3209	0.0004
AISI	0.5629	0.5254	0.6004	266.5817	0.5263	0.6007	0.0067
SIRI	0.6119	0.5759	0.6479	1.2977	0.6344	0.5709	0.5801

AUC, area under the curve.

95% CI, 95% confidence interval.

ED, erectile dysfunction.

CED, complete erectile dysfunction.

## Discussion

Ultimately, the study included 2,128 participants from the 2001–2004 NHANES cohort, of whom 648 (30.45%) had ED. Of the patients with ED, 268 (12.59%) were diagnosed with complete ED. Our findings indicate a negative association between CALLY and ED prevalence, with this association remaining stable in patients with complete ED. The negative correlation persists when CALLY is divided into tertiles (Q1–Q3). Furthermore, subgroup analysis demonstrated that the relationship between CALLY and ED was consistent across all stratifying variables, with a stable negative correlation. Finally, ROC analysis indicated that CALLY may be a superior predictor of ED and complete ED compared to other inflammatory markers. Our study supports previous findings suggesting that inflammation may be a potential mechanism underlying ED. Therefore, incorporating CALLY into clinical practice may help identify individuals at higher risk of ED in the general population.

This is the first study to report CALLY levels in ED patients and controls from the NHANES database. The CALLY index, a novel inflammatory marker, comprises albumin levels, lymphocyte count, and CRP (14). Unlike more straightforward inflammatory indicators like NLR or SII, the CALLY index integrates nutritional, inflammatory, and immune factors to assess inflammation’s impact on erectile dysfunction comprehensively. Albumin, a key indicator of nutritional status, has been implicated in the pathogenesis of ED due to its association with malnutrition (15, 16). Lymphocytes and C-reactive protein (CRP) indicate the body’s immune and inflammatory status. Numerous studies have supported the association between inflammation and ED. Zhong’s analysis revealed that ED patients exhibited higher systemic immune-inflammatory indices(SII) (17). Feng et al. identified a significant positive association between a novel inflammatory marker, the neutrophil-to-lymphocyte ratio (NLR) exceeding 1.52, and ED (18). A study among dialysis patients demonstrated a significant association between C-reactive protein (CRP)/albumin ratio (CAR) and severe ED (19).

However, the mechanisms underlying this association are still not well understood. The most plausible mechanism involves endothelial dysfunction, which ultimately results in arteriosclerosis. Normal vascular endothelium regulates hemostasis, inflammation, and local injury responses, maintaining steady blood flow to mitigate inflammation (20). However, disrupted homeostasis, marked by increased oxidative stress and inflammation, can precipitate endothelial dysfunction (21). Albumin possesses antioxidant properties that can mitigate cell damage caused by free radicals, thereby potentially inhibiting the progression of atherosclerosis. By mitigating the inflammatory response, albumin may help slow the formation of arterial plaques (22). Studies have demonstrated that the presence and severity of endothelial dysfunction correlate with inflammatory markers and mediators like C-reactive protein (23, 24). CRP levels, assessed via penile Doppler ultrasound, significantly correlate with the severity of penile arterial disease (25). Arteriosclerosis uniformly affects blood vessels, and given the smaller diameter of penile arteries (1-2 mm), similar degrees of endothelial dysfunction and arteriosclerosis likely result in substantial reductions in penile tissue blood flow, leading to erectile dysfunction. The pathogenesis of immune cells in erectile dysfunction (ED) is highly complex. Several studies have demonstrated that regulatory B cells expressing CD20 are crucial in suppressing excessive immune activation and inflammation. Regulatory T cells (Tregs) are pivotal in maintaining immune homeostasis and controlling excessive inflammatory responses, thereby safeguarding vascular health—particularly in conditions closely related to vascular function, such as ED (26).

Our study boasts several strengths, including extensive, representative NHANES sample data, fully accounting for sample design and weighting. Additionally, we adjusted for relevant covariates in our multivariate logistic regression analysis to isolate the independent effect of CALLY on erectile dysfunction (ED). Furthermore, subgroup analyses were conducted to examine the stability of these effects. However, our study is not without limitations. For instance, being a cross-sectional study, it cannot establish causality. In addition, due to the limitations of the NHANES database, reliance on self-reported ED history may introduce bias, potentially underestimating the true prevalence of ED. Incorporating objective measures, such as the International Index of Erectile Function (IIEF) or colour Doppler ultrasound, could help mitigate this bias and provide a more accurate assessment.Additionally, while we made efforts to adjust for as many covariates as possible, there remain uncontrolled factors, such as sex hormones, that may still influence the results. Finally, the NHANES database solely represents the United States population. The applicability of the CALLY-ED relationship in other countries or regions requires further verification through additional research. Therefore, future large-scale prospective studies are essential to elucidate the longitudinal relationship between the CALLY index and ED, and clinical data-based studies are needed to assess its predictive and practical value fully.

## Conclusions

This study underscores a significant negative correlation between the CALLY index and the risk of erectile dysfunction (ED). The CALLY index has proven to be a robust and independent predictor of ED, surpassing many other inflammatory markers in performance. This finding emphasizes the potential utility of the CALLY index as a clinical tool for identifying individuals at risk of ED.

## Data Availability

Publicly available datasets were analyzed in this study. This data can be found here: The data used in this study is publicly available from the National Health and Nutrition Examination Survey (NHANES). The dataset can be accessed directly through the following link: https://wwwn.cdc.gov/nchs/nhanes/default.aspx.
