# Supplementary figures and images for: Novel indicator for erectile dysfunction: the CALLY index, evidence from data of NHANES 2001-2004
Source: Front Endocrinol (Lausanne). 2025 Mar 3;16:1527506. doi: 10.3389/fendo.2025.1527506 (PMC11911170; doi:10.3389/fendo.2025.1527506)

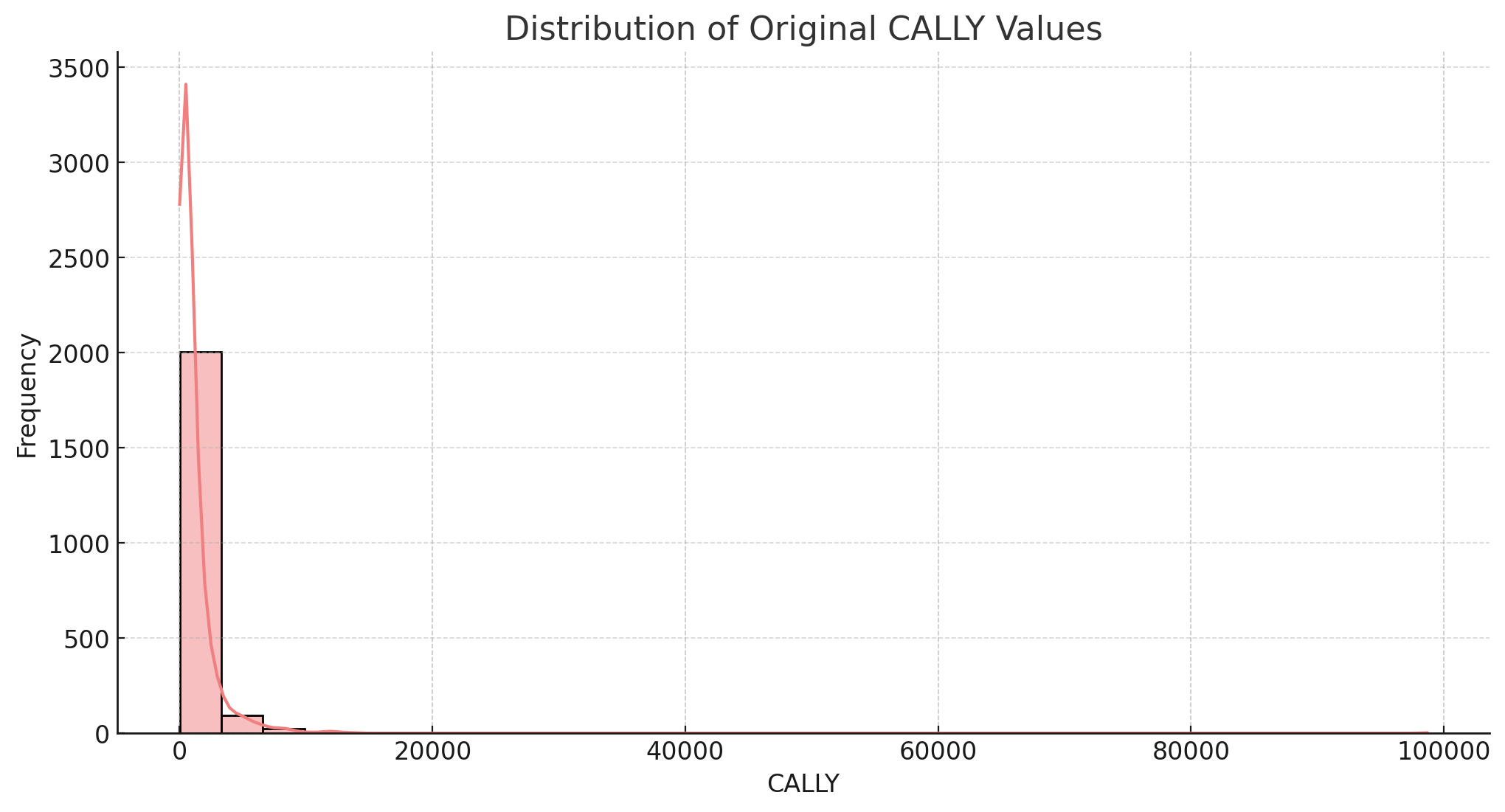

Supplement: Supplementary file 1 [file Image1.png]

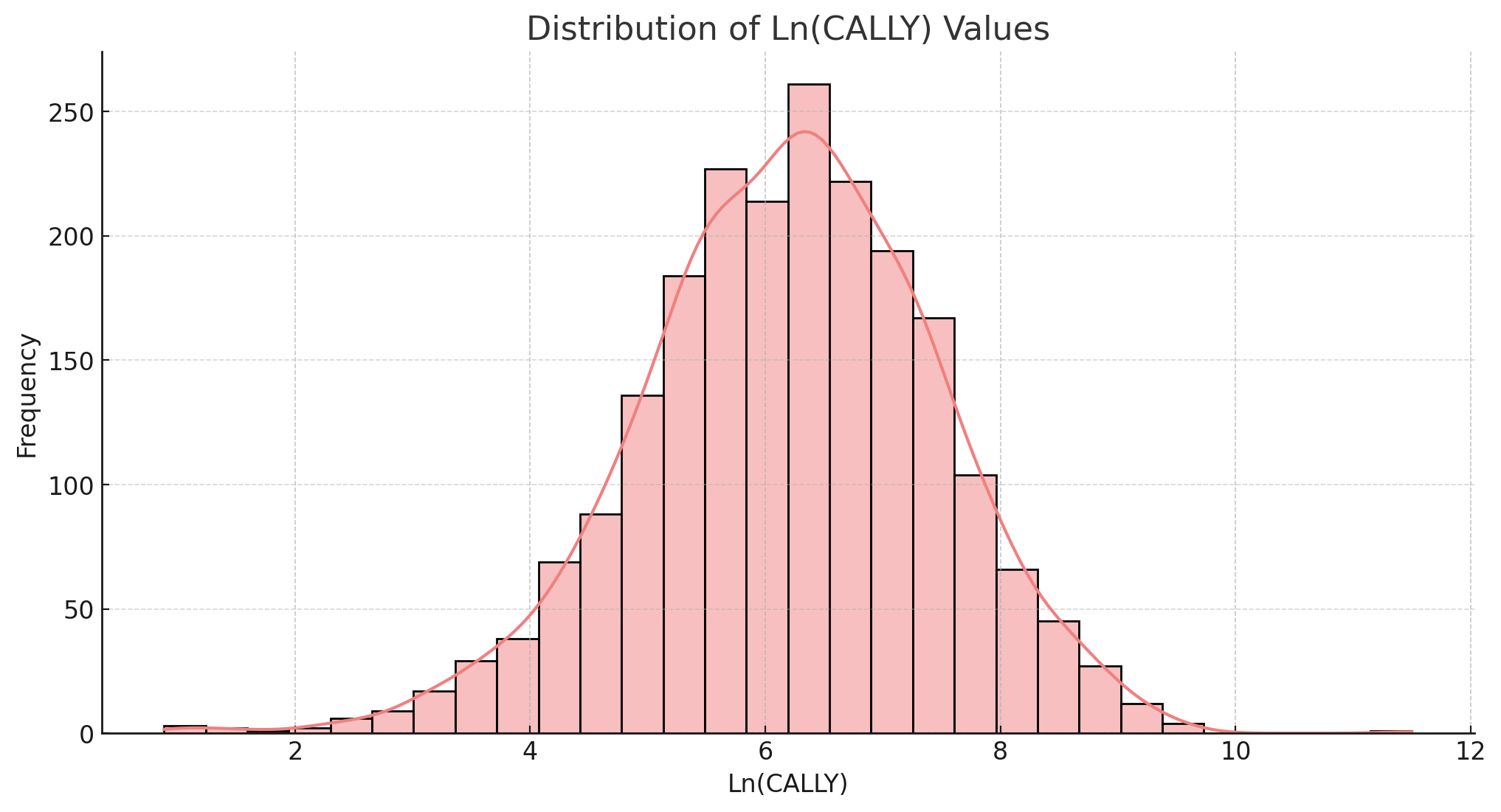

Supplement: Supplementary file 2 [file Image2.png]
